# Math achievement is important, but task values are critical, too: examining the intellectual and motivational factors leading to gender disparities in STEM careers

**DOI:** 10.3389/fpsyg.2015.00036

**Published:** 2015-02-17

**Authors:** Ming-Te Wang, Jessica Degol, Feifei Ye

**Affiliations:** University of Pittsburgh, Pittsburgh, PAUSA

**Keywords:** gender gap, STEM, math achievement, career choice, motivation

## Abstract

Although young women now obtain higher course grades in math than boys and are just as likely to be enrolled in advanced math courses in high school, females continue to be underrepresented in some Science, Technology, Engineering, and Mathematics (STEM) occupations. This study drew on expectancy-value theory to assess (1) which intellectual and motivational factors in high school predict gender differences in career choices and (2) whether students’ motivational beliefs mediated the pathway of gender on STEM career via math achievement by using a national longitudinal sample in the United States. We found that math achievement in 12th grade mediated the association between gender and attainment of a STEM career by the early to mid-thirties. However, math achievement was not the only factor distinguishing gender differences in STEM occupations. Even though math achievement explained career differences between men and women, math task value partially explained the gender differences in STEM career attainment that were attributed to math achievement. The identification of potential factors of women’s underrepresentation in STEM will enhance our ability to design intervention programs that are optimally tailored to female needs to impact STEM achievement and occupational choices.

## INTRODUCTION

Although girls now obtain higher course grades in math than boys and are just as likely to be enrolled in advanced math courses in high school, females continue to be underrepresented in some Science, Technology, Engineering, and Mathematics (STEM) occupations (National Science Foundation, 2011). For example, in 2010 among employed individuals whose highest degree was a Bachelor’s, females comprised around 42% of the workforce in mathematics, 11% of the workforce in engineering, 23% of the workforce in computer and information sciences, and 34% of the workforce in physical sciences (National Science Foundation, 2014).

Career aspirations based on individual competencies, values, and perceived compatibility of competencies and values, are formulated in adolescence and shape the academic pathways that lead to the STEM pipeline (Tai et al., 2006). It is very difficult to initiate a STEM trajectory after beginning college, due to the very constrained and prescribed curricula in STEM fields (Tyson, 2011). Therefore, in order to prevent many talented and capable young women from opting out of the STEM pipeline, it is important to identify the intellectual and psychological factors that surface in the elementary and secondary school years and predict later career choice (Maltese and Tai, 2011; Ceci et al., 2014). In turn, our ability to design intervention programs to impact STEM achievement and occupational choices through these factors will be more optimally tailored to females.

Despite many researchers dedicating themselves to studying the gender gap in STEM fields, the extant literature is limited in several ways. Current reform efforts primarily focus on improving students’ exposure to and performance in advanced-level math courses in high school as a way to address the gender gap in STEM. While encouraging math achievement and enrollment in advanced courses is an important step in setting the foundation for the successful attainment of STEM careers, it alone does not account for the complex motivational factors that influence STEM career choice (Eccles, 2009). In fact, neither mathematical aptitude, nor advanced math course enrollment are strongly predictive of student enjoyment in math-related activities or career choice (Wang and Degol, 2013). Instead, students’ motivational beliefs (e.g., competence beliefs, attitudes, values, interest) about math learning are more critical determinants of future educational and career choices (Maltese and Tai, 2010, 2011). While the importance of motivational beliefs has been widely recognized, most studies are limited to STEM performance or college major as the outcome and very few longitudinal studies have addressed the underlying factors in the high school years that motivate girls to pursue actual STEM careers in adulthood (Lubinski and Benbow, 2006).

Although ability self-concept (feeling competent to succeed) has been shown to be an important predictor of academic performance (Guiso et al., 2008; Hill et al., 2010), personal interest and perceived task value play highly important roles in shaping individual achievement and career choices, and can be more influential than academic self-concept (Eccles, 2009). For example, studies show that in early adolescence, girls and boys tend to endorse different work preferences and lifestyle values (Ferriman et al., 2009). These personal interests and task values can rest outside of students’ perceptions of their own intellectual abilities, and may contribute to the gender gaps in STEM performance and career choices. However, it is unclear whether students’ motivational beliefs (subjective task values in particular) mediate the relation between gender and STEM career through math achievement.

In this study, we draw on Eccles’ (2009) expectancy-value theory to assess which intellectual competencies and motivational beliefs move individuals toward or away from STEM careers. Expectancy-value theory posits that achievement-related choices, such as occupation selection, are most directly influenced by intellectual competencies, ability self-concepts, and the subjective task value attached to the various options. Subjective task value is comprised of interest value (liking or enjoyment), utility value (the instrumental value of the task for helping to fulfill personal goals), attainment value (the link between the task and one’s sense of self, identity, and core personal values), and cost (what may be given up by making a specific choice). Career choices are ultimately made after a number of options, and their various components (e.g., money, authority, social connection) are evaluated and identified as either fitting personal goals or not. Gender differences in career choices reflect gendered differences in relative intellectual competencies, ability self-concepts, and the relative subjective task value of each option under consideration.

### INTELLECTUAL COMPETENCIES

There are small average gender differences between boys and girls on some indicators of intellectual competencies: girls outperform boys in some tests on verbal skills (Park et al., 2008); and girls earn slightly higher grades in all school subjects, including high school math and science (Hyde et al., 2008). Furthermore, differences in the proportion of males and females scoring in the extreme right tail of high stakes math and reading standardized tests have been consistently detected. Males outnumber females in the top 0.01% of the distribution in the SAT and ACT math subtests by 4:1 and 3:1, respectively, while females have a slight advantage on the verbal subtests (Wai et al., 2012). These findings lead to the conclusion that intellectual aptitude, at least by itself, is not the dominant factor in the underrepresentation of women in STEM fields (Ceci and Williams, 2010).

### ABILITY SELF-CONCEPTS

Expectations for success, confidence in one’s abilities to succeed, and personal efficacy have emerged as important predictors of academic achievement and activity involvement (Wigfield et al., 2006). Both boys and girls who rate their math competence highly are more likely to enroll in advanced math courses and receive higher grades in math (Pajares, 2005). Additionally, high school girls tend to rate their math competence lower than boys with similar math grades (Correll, 2001); a finding of particular interest given that poor math self-concept or perceived competence may play a role in female underperformance in mathematics (Durik et al., 2006). Yet intellectual competencies or competence beliefs are a necessary—but not sufficient—predictor of career choices (Joyce and Farenga, 2000). As suggested by expectancy-value theory, career choices depend not only on confidence in one’s abilities to succeed, but also on subjective task values—the value one attaches to relevant subject domains and the goals associated with these domains.

### SUBJECTIVE TASK VALUES

Research on subjective task values shows a number of potentially interrelating effects and gender variations. For instance, despite similarities in math performance, girls’ ‘liking’ of math decreases on average as they move through adolescence to a greater extent than boys’ (Koller et al., 2001). Girls also are more likely to express greater interest in English than math when compared to boys (Jacobs et al., 2002). These findings, in combination with research showing that even females with high math-aptitude tend to express less interest in math-intensive careers (Lubinski and Benbow, 2006), suggest that differential interest and task value in math may contribute to the underrepresentation of women in STEM fields.

Gender differences in occupational and lifestyle values (forms of utility and attainment values) are also potentially important contributing factors to women’s underrepresentation in STEM fields (Lubinski et al., 2001; Ferriman et al., 2009). For example, females are typically more interested in socially oriented careers, while males are more interested in working with objects (Su et al., 2009; Diekman et al., 2011). Meanwhile, women are more likely to value the development of altruistic, reciprocal relationships more than men (Schwartz and Rubel, 2005). This phenomena is illustrated by the fact that women tend to put more value on jobs that allow them to help others and make meaningful contributions to society (communion/affiliative orientation; Abele and Spurk, 2011) and math-intensive careers are usually viewed as being object-oriented (Webb et al., 2002) and less social (Hill et al., 2010).

Finally, research on how priorities beyond career fulfillment help shape females’ decisions to refrain from entering STEM fields, indicate that life values and ‘sense of fit’ are important factors. Per Hakim (2006), women tend to prefer more home-centered lifestyles, whereas men tend to prefer more work-committed lifestyles, and math-related careers are not perceived by females as accommodating to their desired work-family balance. Because work-family balance is highly relevant to career-aged women, most studies have been conducted with adult females; however, this gap in the literature makes it unclear whether family work balance is an important predictor of career choices for high school students.

The current study investigates (1) which intellectual and motivational factors in high school predict gender differences in career choices and (2) whether students’ motivational beliefs mediated the pathway of gender on STEM career through math achievement. Two sets of analyses were conducted to this end. In the first set of analyses, we used hierarchical logistic regression to test whether math ability self-concept and subjective task values (i.e., math interest, social and family values, and desired job characteristics) at 12th grade predicted gender differences in the selection of STEM vs. non-STEM careers, while holding math and reading ability, and family socioeconomic status constant. In the second set of analyses, we tested the role of subjective task values and math achievement as potential mediators for predicting gender differences in selecting STEM and non-STEM careers.

## MATERIALS AND METHODS

### PARTICIPANTS

We used data from the Longitudinal Study of American Youth, a large-scale national study initiated in 1987 that followed two cohorts of students through middle school, high school, and at various stages beyond high school, focusing predominantly on student, family, and school characteristics that influence student achievement, interest, and occupational proclivities toward math and science. The base-year sample consisted of 3,116 students in the 7th grade (mean age = 12 years, cohort II) and 2,829 10th graders (mean age = 15 years, cohort I) from 50 public school systems across the country. Schools were classified as urban (25%), suburban (42%), and rural (33%). Selected schools are considered representative of secondary schools across the country. Each year participants were given standardized tests of math achievement in addition to completing questionnaires about their experiences and attitudes on STEM-related learning. Reading achievement was also assessed for both cohorts in 12th grade. In 2007, when the original study participants were between 33 and 37 years of age, a sample of 3,689 original participants (76% response rate) completed the telephone interview surveys, updating their educational and occupational history from post high school into their mid-thirties. Data used in this study were mainly from two waves: 12th grade and the 2007 follow-up, 14 or 17 years postsecondary school, depending on the cohort. At 12th grade, 75% were White, and 49% were female adolescents.

To determine whether the students who participated in 12th grade differed from those who dropped out between the ages of 33–37, a series of independent samples contingency table analyses and *t*-tests were conducted with all independent, outcome, and demographic variables at 12th grade. Results revealed that those who dropped out of the study did not differ from those who participated in the study at 12th grade. We used full information maximum likelihood (FIML) estimation in Mplus 7.3 to account for missing data in all analysis, as FIML was recommended as the most appropriate approach to handle missing data when data are missing at least at random (Allison, 2012).

### MEASURES

#### STEM occupation

Participants’ occupations at ages 33–37 were self-reported in a telephone interview conducted in 2007. We operationalized occupations into two categories: (1) non-STEM, consisting of careers in the fine arts, literature, business, education, and social sciences, and (2) STEM jobs, consisting of occupations in mathematics, engineering, computer science, life science, medical science, and physical science.

#### Math and reading achievement

Standardized math scores were used from tests taken by students in the spring of 12th grade. The test was developed by the (National Assessment of Educational Progress [NAEP], 1986) to measure students’ knowledge of math, the application and utilization of math knowledge, and integration of math knowledge. A standardized test of reading developed by the Educational Testing Service was used to measure students’ reading comprehension in the spring of 12th grade. Multiple-group item-response theory (IRT) methods were used to scale scores on a metric with a mean of 50 and a SD of 10 (Miller and Kimmel, 2010).

#### Math ability self-concept

Students completed a survey in the fall of 12th grade indicating their math ability self-concept. The math ability self-concept scale (Bleeker and Jacobs, 2004) included three items that measured students’ perceived abilities and expectancy for success in math (e.g., “I am good at math,” “I usually understand math”). The academic self-concept scale was rated from 1 (*strongly disagree*) to 5 (*strongly agree*) with higher scores reflecting higher math ability self-concept (α = 0.80).

#### Subjective task values

In the fall of 12th grade, we measured students’ interest values, utility values, and attainment values (Eccles et al., 1997):

***Math task value.*** The math task value scale included five items that measured students’ interest, enjoyment, and the value they attach to math (e.g., “I enjoy math,” “Math is useful in everyday problems”). The math task value scale was rated from 1 (*strongly disagree*) to 5 (*strongly agree*) with higher scores reflecting higher task values in math (α = 0.75).

***Altruism, family values, and monetary values.*** Students rated the relative importance (1 = *not important*; 2 = *somewhat important*; 3 = *very important*) of a variety of future economic, social, and familial goals. Three separate constructs were generated indicating the extent to which youth exemplified altruistic values, family values, and monetary values. A total of four items were used to indicate the importance students attributed to having an active role in helping others in their communities, including changing social/economic wrongs, staying current on social issues, and helping others within the community (α = 0.77). Family values were constructed using two items that reflected the importance students attributed to having children and prioritizing their family life in the future (α = 0.69). Finally, monetary values measured the extent to which students valued making lots of money in the future. Higher scores indicate placing greater importance on altruism, family values, or monetary values.

***Work preferences.*** Students completed a survey indicating qualities of a future career they would find preferable. Students checked a box to indicate whether they preferred a job with the characteristics listed (1 = *yes*; 0 = *no*). In order to examine the extent to which youth preferred to work with people or objects, two items were examined for the absence or presence of a checkmark: prefers a job that allows work with other people in teams, and prefers a job that allows work with numbers and formulas. Working with teams, therefore, represents a people-oriented job focus and working with numbers and formulas represents an object-oriented job focus.

#### Covariates

We controlled for several potential confounds related to individual career choices in STEM fields, including child gender (0 = *female*; 1 = *male*), child race/ethnicity (0 = *White*; 1 = *others*), parent education (0 = *some college/HS or less*; 1 = *BA/BS or higher*), and parent STEM employment (0 = *parents do not work in STEM or technical field*; 1 = *at least one parent is employed in a STEM or technical profession*). Parental education and employment were collected from parent reports.

## RESULTS

We compared males and females on career choice, covariates, and independent variables. Chi-square tests were used for dichotomous variables, and independent sample *t*-tests for continuous variables (see **Table 1**). More males chose STEM careers than females, and preferred to work with numbers. Moreover, males had higher math achievement, math ability self-concept, math task value, and a greater preference for high-paying careers. In contrast, females had higher reading achievement, altruism, and family values.

**Table 1:** Descriptive statistics of the sample and tests of the difference between female and male (*N* = 5,945).

	Female	Male	*p*-value
**Dependent variable**
Science, Technology, Engineering, and Mathematics (STEM) career (0 = no; 1 = yes; %)	8	10	0.005
Covariates			
Child race (0 = white/other; 1 = black/hispanic; %)	21	21	n.s.
Parent education (0 = some college/HS or less; 1 = BA/BS or higher; %)	29	31	n.s.
Parent STEM occupation (0 = non-STEM; 1 = at least one parent in STEM occupation; %)	17	16	n.s.
Math achievement score	67.24 (12.60)	69.48 (14.60)	<0.001
Reading achievement score	55.34 (27.30)	51.44 (29.60)	<0.001
Math ability self-concept	9.88 (2.85)	10.47 (2.34)	<0.001
Math task value	17.61 (3.80)	18.01 (3.56)	0.002
Altruism	8.13 (1.98)	7.96 (2.03)	0.013
Family values	5.25 (0.97)	4.98 (1.07)	<0.001
Monetary importance	2.23 (0.59)	2.41 (0.61)	<0.001
Prefer job: work with others (0 = no; 1 = yes; %)	53	51	n.s.
Prefer job: work with numbers (0 = no; 1 = yes; %)	14	20	<0.001

Independent sample *t*-test was used for continuous variables and Chi-square tests were used for binary variables. SD are in parentheses.

We conducted hierarchical logistic regression to examine which intellectual and motivational factors were predictive of STEM careers and contributed to gender disparities in selection of STEM occupations, controlling for child gender and race, parent education, and parent STEM employment (see **Table 2**). All continuous predictors, including math achievement, reading achievement, altruism, family values, math ability self-concept, and math task value, were standardized to have mean of zero and SD of one. All dichotomous predictors were indicator coded. Variance Inflation Factor (VIF) was calculated for all predictors and there was no concern with multicollinearity (VIFs < 2.3).

**Table 2:** Hierarchical logistic regression to predict the choice of a STEM occupation.

Predictor	Step 1		Step 2		Step 3		Step 4		Step 5		Step 6		Odds ratio
Gender (1 = male)	0.32	(0.12)**	0.31	(0.12)**	0.16	(0.12)	0.12	(0.13)	0.14	(0.13)	0.12	(0.13)	1.12
Child race (1 = black/hispanic)			-0.69	(0.21)**	-0.22	(0.22)	-0.35	(0.22)	-0.29	(0.23)	-0.31	(0.23)	0.74
Parent education			0.78	(0.12)***	0.44	(0.13)***	0.53	(0.13)***	0.55	(0.13)***	0.56	(0.13)***	1.75
Parent STEM occupation			0.33	(0.14)*	0.18	(0.14)	0.17	(0.15)	0.18	(0.15)	0.15	(0.15)	1.17
Math achievement score					0.79	(0.12)***	0.64	(0.12)***	0.66	(0.12)***	0.65	(0.13)***	1.91
Reading achievement score					0.02	(0.09)	0.01	(0.09)	0.02	(0.09)	0.02	(0.10)	1.02
Motivational beliefs and values													
Math ability self-concept							0.11	(0.10)	0.08	(0.10)	0.07	(0.11)	1.07
Math task value							0.40	(0.09)***	0.44	(0.09)***	0.42	(0.09)***	1.53
Altruism									-0.20	(0.08)*	-0.20	(0.08)*	0.82
Family values									0.09	(0.08)	0.08	(0.08)	1.08
Monetary importance									-0.08	(0.07)	-0.08	(0.07)	0.92
Prefer job: work with others											0.28	(0.15)	1.32
Prefer job: work with numbers											0.26	(0.17)	1.29

All continuous predictors were standardized, including math achievement score, reading achievement score, math ability self-concept, math task value, altruism, family values, and monetary importance. *p < 0.05; **p < 0.01; ***p < 0.001.

In the first set of hierarchical logistic regression models, we included gender as the only predictor to show the gender disparity in STEM occupation. Males were 1.38 times (*p* = 0.005) as likely as females to choose STEM careers. Second, we added student race/ethnicity, parent education, and parent STEM occupation, which all significantly predicted STEM occupation (*p*s < 0.02). The gender effect was still significant, but the odds ratio decreased to 1.36 (*p* = 0.009). Third, we added math and reading achievement, and found that only math achievement was significantly related to STEM occupation. Importantly, the gender effect was reduced to non-significance. Fourth, we added math ability self-concept and math task value, in which only math task value was positively associated with STEM occupation. Fifth, we added altruism, family values, and monetary importance, with only altruism negatively predicting STEM occupation. Finally, we added student’s work preferences (e.g., either working with people or objects), both of which failed to significantly differentiate career choices.

**FIGURE 1:**
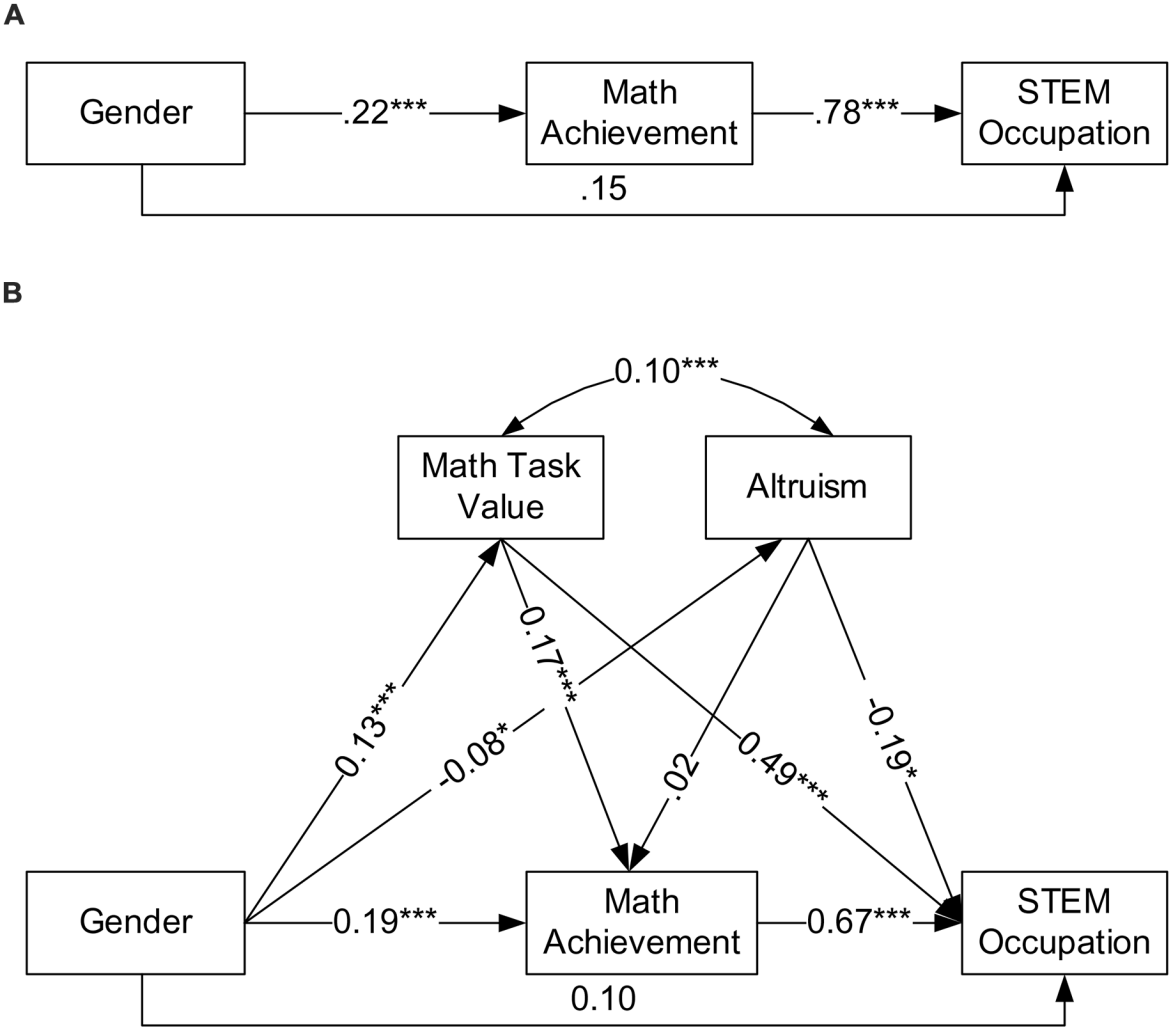
**Mediation effects of math task value, altruism, and math achievement on gender difference in STEM occupation. (A)** Presents results with math achievement as the only mediator and **(B)** presents results with math task value and altruism added as mediators in addition to math achievement. All coefficients are unstandardized, adjusted with covariates of race, parent education, parent occupation, and reading achievement. *p < 0.05; ***p < 0.001.

In order to test the mediation effect of math task value and math achievement on gender differences in career choice, we adopted the outlined procedure in Baron and Kenny (1986). We first assessed the total direct effect of gender on STEM occupation with a logistic regression model while partialling out the effects of such covariates as race, parent education, parent occupation, and reading achievement from STEM occupation. Then we conducted two path models to tease out the mediation effects of math achievement, math task values, and altruism on gender difference in STEM occupation, while partialling out the effects of the covariates from all mediators and STEM occupation. In the first mediation model (**Figure 1A**), we tested only the mediation effect of math achievement. In the second mediation model (**Figure 1B**), we added math task values and altruism as additional mediators given that the hierarchical logistic regression results suggested that altruism and math task values were the only significant motivational predictors of STEM occupation. A direct relationship was modeled from math task value and altruism to math achievement. Then the indirect effects were tested using bias-corrected and accelerated (BCa) bootstrap confidence interval (BCI).

The total direct effect of gender on STEM occupation was significant, *B* = 0.36, *p* = 0.002, odds ratio = 1.43. With math achievement as the only mediator in the model (**Figure 1A**), the direct effect from gender to STEM occupation became not significant, *B* = 0.15, *p* = 0.21, odds ratio = 1.17. When math task value and altruism were added as mediators in addition to math achievement (**Figure 1B**), the direct effect was further reduced to *B* = 0.10, *p* = 0.41, odds ratio = 1.11. The relative indirect effect (Huang et al., 2004), loosely interpreted as the proportion of the total effect that is mediated, was calculated to be 1-0.15/0.36 = 0.58 with math achievement as the mediator, and 1-0.10/0.36 = 0.72 with math achievement, math task value, and altruism as the mediators.

**Table 3** presents indirect effects (unstandardized path coefficients) and their BCa BCI. In the first path model, math achievement significantly mediated the gender difference in STEM occupation, with the indirect effect estimated to be 0.17 (95% BCI: 0.11-0.24), indicating that, indirectly via math achievement, the odds of males choosing STEM occupations increased by 1.19 times that of females. In the second path model, males had higher math achievement and math task value, but lower altruism. Math task value was significantly associated with math achievement, while altruism was not. Both math task value and altruism were directly and significantly associated with STEM occupation, positively for math task value, and negatively for altruism. We found four significant indirect paths, including: (1) Gender → Math Task Value → STEM occupation, (2) Gender → Altruism → STEM occupation, (3) Gender → Math Task Value → Math Achievement → STEM occupation, and (4) Gender → Math Achievement → STEM occupation. The relative indirect effect indexes were 0.17, 0.06, 0.06, and 0.36 for these four significant indirect effects. It is noteworthy that math task value not only mediated the gender difference in STEM occupation, but its positive relationship with math achievement also accounted partially for the mediating effect of math achievement on the gender effect. By including math task value and altruism, the indirect effect of Gender → Math Achievement → STEM Occupation was reduced from 0.17 to 0.13, with the corresponding relative indirect effect index reduced from 0.58 to 0.36. However, the magnitude of the Gender → Math Task Value → Math Achievement → STEM occupation effect has a small value of 0.02. This is not surprising given that the effects of race, parent education, parent occupation, and reading achievement were controlled for with all mediators. Results showed that math achievement was significantly related with gender (*B* = 0.19, *p* < 0.001), race (*B* = -0.43, *p* < 0.001), parent education (*B* = 0.23, *p* < 0.001), parent occupation (*B* = 0.13, *p* = 0.001), and reading achievement (*B* = 0.54, *p* < 0.001). Math task value was significantly related with gender (*B* = 0.13, *p* < 0.001), race (*B* = 0.25, *p* < 0.001), and reading achievement (*B* = 0.15, *p* < 0.001).

**Table 3:** Mediation effect of math achievement, task value, and altruism on gender difference in STEM occupation.

	Mediation model 1		Mediation model 2	
	Estimate	Bootstrap 95% CI	Estimate	Bootstrap 95% CI
Indirect path				
Gender → math task value → STEM occupation			0.06	(0.03, 0.11)
Gender → altruism → STEM occupation			0.02	(0.002, 0.04)
Gender → math task value → math achievement → STEM occupation			0.02	(0.006, 0.03)
Gender → altruism → math achievement → STEM occupation			0.001	(-0.005, 0.000)
Gender → math achievement → STEM occupation	0.17	(0.11, 0.24)	0.13	(0.08, 0.19)

## DISCUSSION

Increasing opportunities for female participation in STEM fields is a pivotal social, economic, and political issue in the advancement of female interests. In order to elucidate the factors associated with females’ underrepresentation in STEM, the current study examined which factors predicted gender differences in the selection of STEM occupations, and whether math task values and altruism mediated the pathway of the gender effect on STEM career choice through math achievement. Identifying potential barriers that keep women from fulfilling their potential in STEM fields will help inform intervention efforts targeting the removal of these barriers.

We found that math achievement in 12th grade mediated the association between gender and attainment of a STEM career by the early to mid-thirties. Women were less likely than men to pursue a career in STEM, but this relation was explained by gender differences in math achievement in high school. Our results show that women, on average, had lower math standardized scores than men, and unsurprisingly, individuals with higher math achievement were more likely to attain a career in STEM. However, math achievement was not the only factor distinguishing gender differences in STEM occupations. Math task value partially mediated the pathways among gender, math achievement, and STEM careers. Women had lower math task values than men, and lower math task value was associated with lower math achievement and lower likelihood of pursuing STEM careers. Essentially, despite math achievement explaining career differences between men and women, math task value also contributed to the gender differences in STEM career attainment that were attributed to math achievement. These findings shed some light onto the complex ways that ability self-concept and subjective task values operate in promoting STEM career selection. Expectancy-value theory posits that individuals consider multiple factors when selecting potential careers, including prior achievement, perceived competencies, and task values. In line with this theory, math task value and altruism (a form of utility value) predicted STEM career, but math ability self-concept did not. Previous research has found that ability self-concept is predictive of academic achievement, but, unlike subjective task values, it is not consistently linked to educational or career choices (Durik et al., 2006). In other words, believing that you are good at a task may further enhance your performance in the task, but it does not mean that you enjoy the task and will continue to pursue it.

How do these findings relate to factors associated with females’ underrepresentation in STEM fields? Increasing math achievement is important for increasing women’s representation in STEM, but achievement alone may not be sufficient. We know that achievement matters for STEM enrollment; many of the most mathematically talented individuals eventually achieve prestigious careers in STEM fields (Wai et al., 2010). Historically, women’s underperformance in quantitative reasoning skills relative to men’s has been considered one of the main factors in women’s decisions to opt out of STEM fields (Halpern, 2007). In response, public focus and political initiatives have centered on increasing female math performance and advanced math course enrollment. However, converging evidence from the current study and other research has demonstrated that increasing quantitative skills alone will not effectively lead to greater female participation in STEM (Ceci and Williams, 2011; Maltese and Tai, 2011; Riegle-Crumb et al., 2012). While gender differences in attainment of STEM careers was explained by lower female performance on standardized math tests in our first model, the second model demonstrated that this pathway (gender to achievement to STEM career) was partially attributed to gender differences in math task values.

Girls consistently express less interest in math (Jacobs et al., 2002) and view math and STEM careers as less aligned with their personal career interests and goals (Su et al., 2009). Studies have shown that greater interest and greater perceived importance and utility value of math may lead to greater investment in and persistence in math activities, which ultimately lead to higher math achievement (Wigfield and Eccles, 2002; Wang, 2012). Therefore, aside from promoting greater math achievement, current policy initiatives also need to target the development of math task values: encouraging interest in math and its utility value. When women see STEM fields as useful, widely applicable, and viable career options they will be more likely to opt into them.

Given that math interest and task values are linked to academic performance, the benefits derived from enhancing math task value may be twofold. If math task values impact math achievement and selection of STEM occupations, then intervening to promote subjective task values in math should not only increase STEM persistence in the long run, but also enhance math achievement. Since math achievement positively predicted long term decisions in STEM career, the developmental impacts of interventions that seek to increase math task value could be exponential compared to programs that target math skills alone. Furthermore, targeting math task values may not only lead to increases in math achievement, but improved math performance may actually further enhance math task value, given that the two are reciprocally linked over time. Exclusively focusing on math achievement as a path to STEM persistence is a unimodal answer, while increasing math task values in addition to achievement is a multimodal solution that could activate multiple pathways to a STEM career.

Furthermore, since our study shows that math task values begin to predict students’ STEM attainment as early as high school, early intervention is vital. Recent studies have shown that interest and career aspirations in STEM emerge prior to entry into high school, and that by 12th grade the decision to major in a STEM vs. non-STEM career is largely solidified for many students (Maltese and Tai, 2011). While there are an increasing number of programs that target student interest, enjoyment, and engagement in STEM (e.g., Detroit Area Pre-College Engineering Program, Great Explorations in Math and Science, Project Lead the Way), these crucial motivating factors should become a greater focus of all k-12 interventions. Particularly, given that increases in STEM course taking and achievement among females have not led to comparable increases in STEM workforce participation, programs need to strengthen teacher training and redesign curriculum to include targeted strategies for dispelling gender stereotypes and increasing female interest in STEM.

Our findings suggest that enhancing women’s math task value may be instrumental in inspiring larger numbers of women to seriously consider STEM fields as viable career options. But how would this look in practice? We know that students are more engaged in classrooms that incorporate hands-on learning, creative thinking, and challenging real-world applications of problems and concepts (Marks, 2000). For girls and women in particular, it may be helpful to take a proactive approach that utilizes their unique strengths. For instance, a recent study showed that girls are more likely than boys to have both high verbal and math skills (Wang et al., 2013). Therefore, incorporating storytelling into math may not only capitalize on the strengths of girls’ verbal skills but also increase female interest in math and science by making these subjects appear hands-on and practical. Additionally, specific teaching strategies such as focusing on women’s historical contributions to these fields, and increasing girls’ exposure and access to female scientists and engineers as career role models (Steinke et al., 2007), may help combat the pervasive math-gender stereotypes that affect girls’ math identities as young as 6 years of age (Cvencek et al., 2011).

Altruism can also be emphasized. Women view helping people and contributing to the greater good as highly important career goals (Su et al., 2009; Abele and Spurk, 2011), which are not perceived to be in line with STEM careers. Indeed, our study suggests that altruism mediated the gender effect on STEM occupations. Since it is plainly not the goal to make women less altruistic, STEM educators should place greater emphasis on demonstrating how female scientists can develop technologies and make discoveries that greatly benefit people’s lives. This is in line with the National Academy of Engineering [NAE]’s (2013) recent efforts to alter public perceptions by communicating that engineering is a helping profession that works on solving problems of human health and safety throughout the world.

Interestingly, differences in family and monetary values, and preferences for working with people or numbers did not explain gender differences in STEM careers. The lack of findings for family values was not unexpected; previous research has demonstrated that gender differences in work/family balance preferences do not typically emerge until the mid-30 s or adulthood, when women are more likely to be raising children and building their families (Ferriman et al., 2009). Since family values were assessed in 12th grade, we can expect that work/family lifestyle preferences will not yet factor prominently in determining male/female differences in STEM choice. Similarly, monetary values may not have predicted gender differences in career choices at this age, because much like family values, concern over earned income is a distal issue that will be experienced more fully in adulthood. More immediate concerns over money, such as tuition costs and student loan debt, may have greater bearing in adolescence. Preferences for working with people or numbers, which typically differ along gender lines, also failed to explain gender differences in STEM careers. Most occupations allow for the opportunity to work with people, numbers, and objects to varying degrees. The key difference is in how prominently these aspects are featured in a career (e.g., interacting directly with people, such as teaching vs. interacting directly with numbers, such as engineering). However, enjoyment of working with numbers does not necessarily indicate a lack of enjoyment in working with people and vice versa, and both may be large components of the same career (e.g., teaching engineering students). For this reason, it is likely that these preferences may not explain gender differences in STEM career selection to the same degree as math task value and altruism.

## CONCLUSION

Despite women’s advances in the U.S. workforce, their entrance into lucrative STEM careers has been less successful, and these professions continue to be heavily male-dominated. The prestige and innovation surrounding math and science, along with their accompanying economic benefits, are not extended to women when they are non-participatory in these fields. Our study builds on well-established literature by identifying the intellectual and motivational factors contributing to women’s underrepresentation in STEM. However, it is important to reiterate that generating greater female interest in STEM should not be equated with forcing unwanted career choices on them. We do not want to coerce women into STEM fields if they have no interest in them, and we do not want to undermine the importance and value of non-STEM careers. Instead, we seek to alter instructional approaches to math and science education to demonstrate how STEM careers can benefit society and provide opportunities for helping and interacting with others, thereby, merging women’s personal task values and career aspirations. Furthermore, many adolescents may not truly understand what it means to obtain a degree in STEM (Fralick et al., 2009). Introducing youth to the different majors they can pursue in STEM and the careers that these degrees will prepare them for can provide adolescents with a better understanding of the nature of these occupations. Ensuring that females are well informed of the full diversity of options available in STEM will enable math-competent females to better evaluate the utility and cost of different STEM careers. Our main goals are to present all of the STEM career opportunities available to women, remove misconceptions that operate as barriers to STEM enrollment, and empower women to make career decisions that best meet their needs for personal and occupational fulfillment.

## Conflict of Interest Statement

The authors declare that the research was conducted in the absence of any commercial or financial relationships that could be construed as a potential conflict of interest.

## References

[B1] Abele A. E. Spurk D. (2011). The dual impact of gender and the influence of timing of parenthood on men’s and women’s careers development: longitudinal findings. *Int. J. Behav. Dev.* 35 225–232 10.1177/0165025411398181

[B2] Allison P. (2012). Handling missing data by maximum likelihood. *Paper 312–2012 presented at SAS Global Forum 2012* Orlando, FL.

[B3] Baron R. M. Kenny D. A. (1986). The moderator-mediator variable distinction in social psychological research: conceptual, strategic, and statistical considerations. *J. Pers. Soc. Psychol.* 51 1173–1182 10.1037/0022-3514.51.6.1173 3806354

[B4] Bleeker M. Jacobs J. E. (2004). Achievement in math and science: do mothers’ beliefs matter 12 years later? *J. Educ. Psychol.* 96 97–109 10.1037/0022-0663.96.1.97

[B5] Ceci S. J. Ginther D. K. Kahn S. Williams W. M. (2014). Women in academic science: a changing landscape. *Psychol. Sci. Public Interest* 15 75–141 10.1177/1529100614541236 26172066

[B6] Ceci S. J. Williams W. M. (2010). Sex differences in math-intensive fields. *Curr. Dir. Psychol. Sci.* 19 275–279 10.1177/0963721410383241 21152367 PMC2997703

[B7] Ceci S. J. Williams W. M. (2011). Understanding current causes of women’s underrepresentation in science. *Proc. Natl. Acad. Sci. U.S.A.* 108 3157–3162 10.1073/pnas.1014871108 21300892 PMC3044353

[B8] Correll S. J. (2001). Gender and the career choice process: the role of biased self-assessments. *Am. J. Soc.* 106 1691–1730 10.1086/321299

[B9] Cvencek D. Meltzoff A. N. Greenwald A. G. (2011). Math–gender stereotypes in elementary school children. *Child Dev.* 82 766–779 10.1111/j.1467-8624.2010.01529.x 21410915

[B10] Diekman A. B. Clark E. K. Johnston A. M. Brown E. R. Steinberg M. (2011). Malleability in communal goals and beliefs influences attraction to STEM careers: evidence for a goal congruity perspective. *J. Pers. Soc. Psychol.* 101 902–918 10.1037/a0025199 21859224

[B11] Durik A. M. Vida M. Eccles J. S. (2006). Task values and ability beliefs as predictors of high school literacy choices: a developmental analysis. *J. Educ. Psychol.* 98 382–393 10.1037/0022-0663.98.2.382

[B12] Eccles J. S. (2009). Who am I and what am I going to do with my life? Personal and collective identities as motivators of action. *Educ. Psychol.* 44 78–89 10.1080/00461520902832368

[B13] Eccles J. S. Lord S. E. Roeser R. W. Barber B. L. Jozefowicz D. M. (1997). “The association of school transitions in early adolescence with developmental trajectories through high school,” in *Health Risks and Developmental Transitions During Adolescence* eds Schulenberg J. Maggs J. I. Hurrelmann K. (New York: Cambridge University Press) 283–321.

[B14] Ferriman K. Lubinski D. Benbow C. P. (2009). Work preferences, life values, and personal views of top math/science graduate students and the profoundly gifted: developmental changes and gender differences during emerging adulthood and parenthood. *J. Pers. Soc. Psychol.* 97 517–532 10.1037/a0016030 19686005

[B15] Fralick B. Kearn J. Thompson S. Lyons J. (2009). How middle schoolers draw engineers and scientists. *J. Sci. Educ. Technol.* 18 60–73 10.1007/s10956-008-9133-3

[B16] Guiso L. Monte F. Sapienza P. Zingales L. (2008). Culture, gender, and math. *Science* 320 1164–1165 10.1126/science.1154094 18511674

[B17] Hakim C. (2006). Women, careers, and work-life preferences. *Br. J. Guid. Couns.* 34 279–294 10.1080/03069880600769118

[B18] Halpern D. F. (2007). “Science sex and good sense: why women are underrepresented in some areas of science and math,” in *Why Aren’t more Women in Science? Top Researchers Debate the Evidence* eds Ceci W. M. Williams S. J. (Washington, DC: American Psychological Association) 121–130 10.1037/11546-010

[B19] Hill C. Corbett C. St. Rose A. (2010). *Why so few? Women in Science, Technology, Engineering, and Mathematics*. Washington, DC: American Association of University Women.

[B20] Huang B. Sivaganesan S. Succop P. Goodman E. (2004). Statistical assessment of mediational effects for logistic mediational models. *Stat. Med.* 23 2713–2728 10.1002/sim.1847 15316954

[B21] Hyde J. S. Lindberg S. M. Linn M. C. Ellis A. B. Williams C. C. (2008). Gender similarities characterize math performance. *Science* 321 494–495 10.1126/science.1160364 18653867

[B22] Jacobs J. E. Lanza S. Osgood D. W. Eccles J. S. Wigfield A. (2002). Changes in children’s self-competence and values: gender and domain differences across grades one through twelve. *Child Dev.* 73 509–527 10.1111/1467-8624.00421 11949906

[B23] Joyce B. A. Farenga S. J. (2000). Young girls in science: academic ability, perceptions, and future participation in science. *Roeper Rev.* 22 261–262 10.1080/02783190009554048

[B24] Koller O. Baumert J. Schnabel K. (2001). Does interest matter? The relationship between academic interest and achievement in mathematics. *J. Res. Math. Educ.* 32 448–470 10.2307/749801

[B25] Lubinski D. Benbow C. P. (2006). Study of mathematically precocious youth after 35 years: uncovering antecedents for the development of math-science expertise. *Perspect. Psychol. Sci.* 1 316–345 10.1111/j.1745-6916.2006.00019.x 26151798

[B26] Lubinski D. Webb R. M. Morelock M. J. Benbow C. P. (2001). Top 1 in 10,000: a 10-year follow-up of the profoundly gifted. *J. Appl. Psychol.* 86 718–729 10.1037/0021-9010.86.4.718 11519655

[B27] Maltese A. V. Tai R. H. (2010). Eyeballs in the fridge: sources of early interest in science. *Int. J. Sci. Educ.* 32 669–685 10.1080/09500690902792385

[B28] Maltese A. V. Tai R. H. (2011). Pipeline persistence: examining the association of educational experiences with earned degrees in STEM among US students. *Sci. Educ.* 95 877–907 10.1002/sce.20441

[B29] Marks H. M. (2000). Student engagement in instructional activity: patterns in the elementary, middle, and high school years. *Am. Educ. Res. J.* 37 153–184 10.3102/00028312037001153

[B30] Miller J. D. Kimmel L. (2010). *Longitudinal Study of American Youth User’s Manual: Student, Parent, and School Data (1987-Continuing)*. Ann Arbor, MI: ICPSR.

[B31] National Academy of Engineering [NAE]. (2013). *Messaging for Engineering: From Research to Action*. Washington, DC: The National Academies Press.

[B32] National Assessment of Educational Progress [NAEP]. (1986). *Math Objectives 1985–1986 Assessment.* Princeton, NJ: Educational Testing Service.

[B33] National Science Foundation. (2011). *Women, Minorities, and Persons with Disabilities in Science and Engineering: 2011*. Arlington, VA.: National Science Foundation.

[B34] National Science Foundation. (2014). *Survey of Doctorate Recipients, 2013. National Center for Science and Engineering Statistics*. Available at: http://ncsesdata.nsf.gov/doctoratework/2013/

[B35] Pajares F. (2005). “Gender differences in mathematics self-efficacy beliefs,” in *Gender Differences in Mathematics: An Integrative Psychological Approach* eds Gallagher A. M. Kaufmann J. C. (Boston, MA: Cambridge University Press) 294–315.

[B36] Park G. Lubinski D. Benbow C. P. (2008). Ability differences among people who have commensurate degrees matter for scientific creativity. *Psychol. Sci.* 19 957–961 10.1111/j.1467-9280.2008.02182.x 19000201

[B37] Riegle-Crumb C. King B. Grodsky E. Muller C. (2012). The more things change, the more they stay the same? Prior achievement fails to explain gender inequality in entry into STEM college majors over time. *Am. Educ. Res. J.* 49 1048–1073 10.3102/0002831211435229 PMC3872126 24371330

[B38] Schwartz S. H. Rubel T. (2005). Sex differences in value priorities: cross-cultural and multi-method studies. *J. Pers. Soc. Psychol.* 89 1010–1028 10.1037/0022-3514.89.6.1010 16393031

[B39] Steinke J. Lapinski M. K. Crocker N. Zietsman-Thomas A. Williams Y. Evergreen S. H. (2007). Assessing media influences on middle school–aged children’s perceptions of women in science using the draw-a-scientist test (DAST). *Sci. Commun.* 29 35–64 10.1177/1075547007306508

[B40] Su R. Rounds J. Armstrong P. I. (2009). Men and things, women and people: a meta-analysis of sex differences in interests. *Psychol. Bull.* 135 859–884 10.1037/a0017364 19883140

[B41] Tai R. H. Liu C. Q. Maltese A. V. Fan X. (2006). Planning early for careers in science. *Science* 312 1143–1144 10.1126/science.1128690 16728620

[B42] Tyson W. (2011). Modeling engineering degree attainment using high school and college physics and calculus course taking and achievement. *J. Eng. Educ.* 100 760–777 10.1002/j.2168-9830.2011.tb00035.x

[B43] Wai J. Lubinski D. Benbow C. P. Steiger J. H. (2010). Accomplishment in science, technology, engineering, and mathematics (STEM) and its relation to STEM educational dose: a 25-year longitudinal study. *J. Educ. Psychol.* 102 860–871 10.1037/a0019454

[B44] Wai J. Putallaz M. Makel M. C. (2012). Studying intellectual outliers: are there sex differences, and are the smart getting smarter? *Curr. Dir. Psychol. Sci.* 21 382–390 10.1177/0963721412455052

[B45] Wang M. T. (2012). Educational and career interests in math: a longitudinal examination of the links between perceived classroom environment, motivational beliefs, and interests. *Dev. Psychol.* 48 1643–1657 10.1037/a0027247 22390667

[B46] Wang M. T. Degol J. (2013). Motivational pathways to STEM career choices: using expectancy-value perspective to understand individual and gender differences in STEM fields. *Dev. Rev.* 33 304–340 10.1016/j.dr.2013.08.001 PMC3843492 24298199

[B47] Wang M. T. Eccles J. S. Kenny S. (2013). Not lack of ability but more choice: individual and gender differences in STEM career choice. *Psychol. Sci.* 24 770–775 10.1177/0956797612458937 23508740

[B48] Webb R. M. Lubinski D. Benbow C. P. (2002). Mathematically facile adolescents with math-science aspirations: new perspectives on their educational and vocational development. *J. Educ. Psychol.* 94 785–794 10.1037/0022-0663.94.4.785

[B49] Wigfield A. Eccles J. S. (2002). “The development of competence beliefs, expectancies for success, and achievement values from childhood through adolescence,” in *Development of Achievement Motivation* eds Wigfield A. Eccles J. S. (San Diego, CA: Academic Press) 91–120.

[B50] Wigfield A. Eccles J. S. Davis-Kean P. Roeser R. Scheifele U. (2006). “Motivation to succeed,” in *Handbook of Child Psychology. Social, Emotional, and Personality Development* Vol. 3 6th Edn eds Damon W. Eisenberg N. (New York: Wiley) 933–1002.

